# Supplementary material for: Small Brown Planthopper Nymph Infestation Regulates Plant Defenses by Affecting Secondary Metabolite Biosynthesis in Rice
Source: Int J Mol Sci. 2023 Mar 1;24(5):4764. doi: 10.3390/ijms24054764 (PMC10003665; doi:10.3390/ijms24054764)
Supplement: Supplementary file 1 [file ijms-24-04764-s001.zip › Figure S1.pdf]

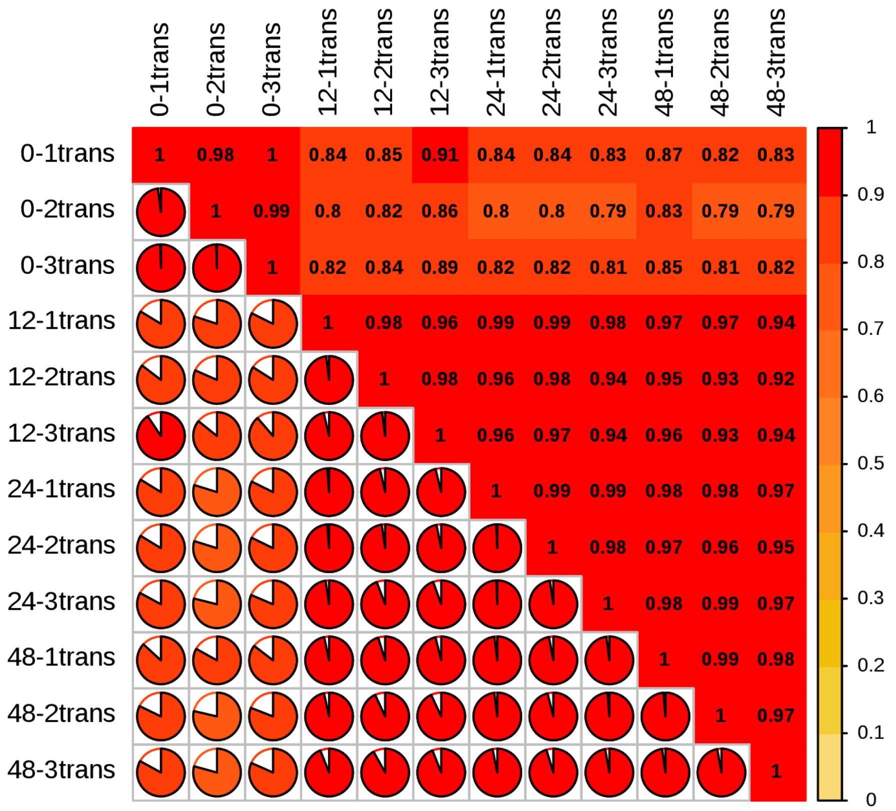

Figure. S1. Gene expression pattern correlation analysis between the biological replicate samples.
