# Supplementary material for: Small Brown Planthopper Nymph Infestation Regulates Plant Defenses by Affecting Secondary Metabolite Biosynthesis in Rice
Source: Int J Mol Sci. 2023 Mar 1;24(5):4764. doi: 10.3390/ijms24054764 (PMC10003665; doi:10.3390/ijms24054764)
Supplement: Supplementary file 1 [file ijms-24-04764-s001.zip › Figure S2.pdf]

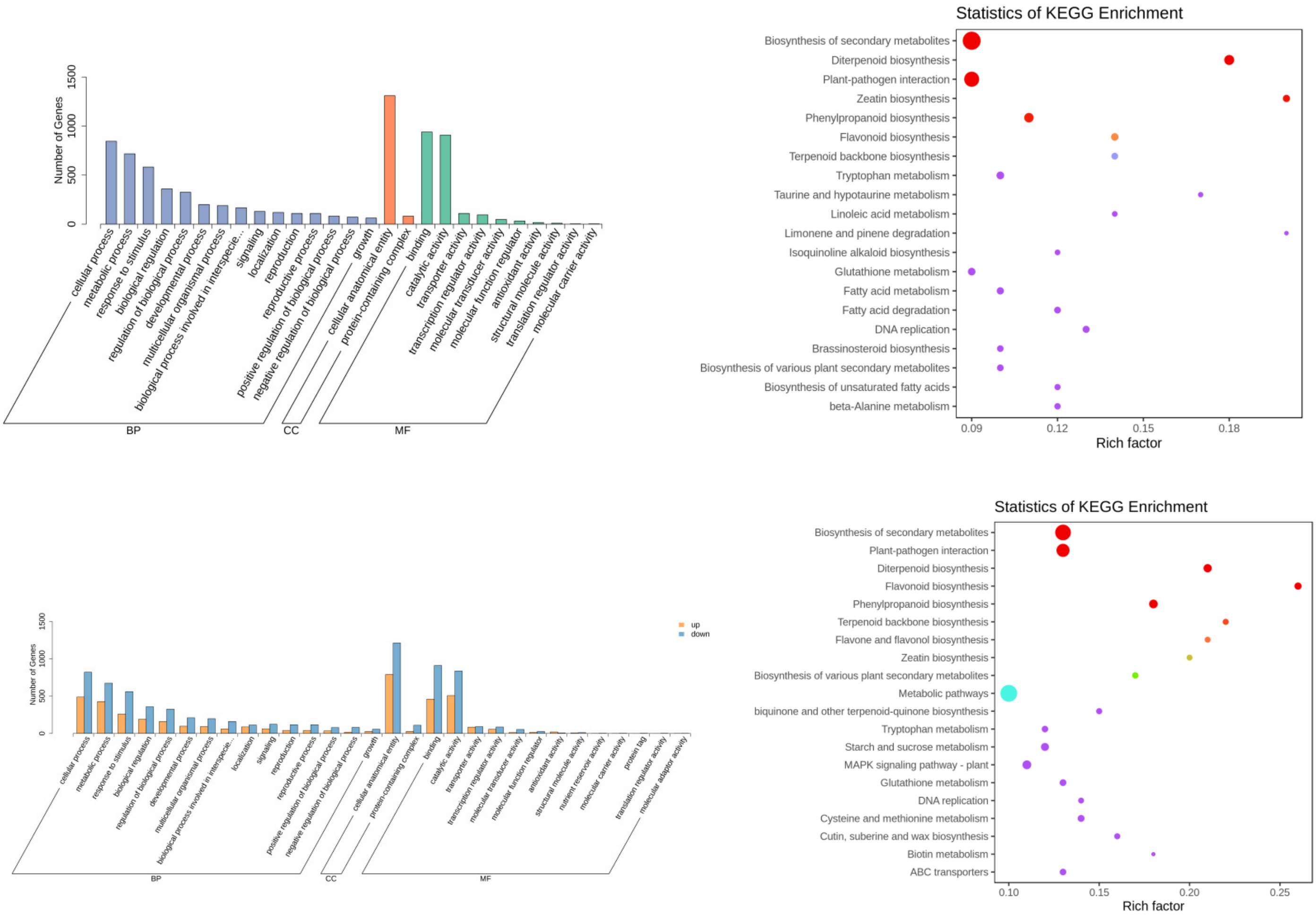

Figure S2. Statistics of Gene Ontology (GO) enrichment (left part) and Kyoto Encyclopedia of Genes and Genomes (KEGG) pathway enrichment (right part) of differentially expressed genes in trans12 vs. trans0 group (upper pictures) and trans48 vs. trans0 group (bottom pictures), respectively. *p*-value ≤ 0.05 and the absolute value of Log2 FC (fold-change) ≥ 1.0 are used as the threshold for significant difference expression.
