# Supplementary material for: Small Brown Planthopper Nymph Infestation Regulates Plant Defenses by Affecting Secondary Metabolite Biosynthesis in Rice
Source: Int J Mol Sci. 2023 Mar 1;24(5):4764. doi: 10.3390/ijms24054764 (PMC10003665; doi:10.3390/ijms24054764)
Supplement: Supplementary file 1 [file ijms-24-04764-s001.zip › Figure S3.pdf]

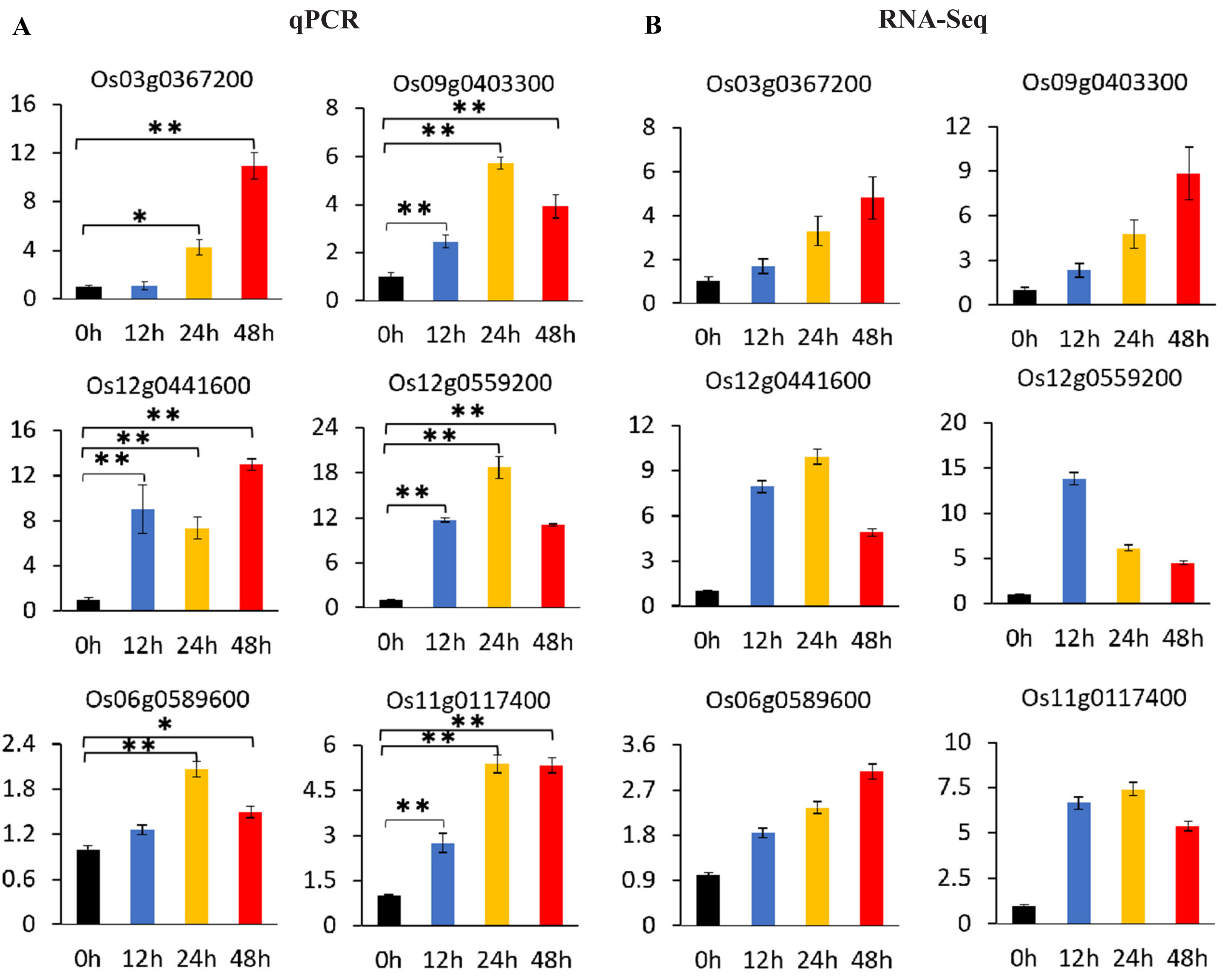

A
qPCR
B
RNA-Seq
Os03g0367200
Os09g0403300
Os12g0441600
Os12g0559200
Os06g0589600
Os11g0117400
0h
12h
24h
48h

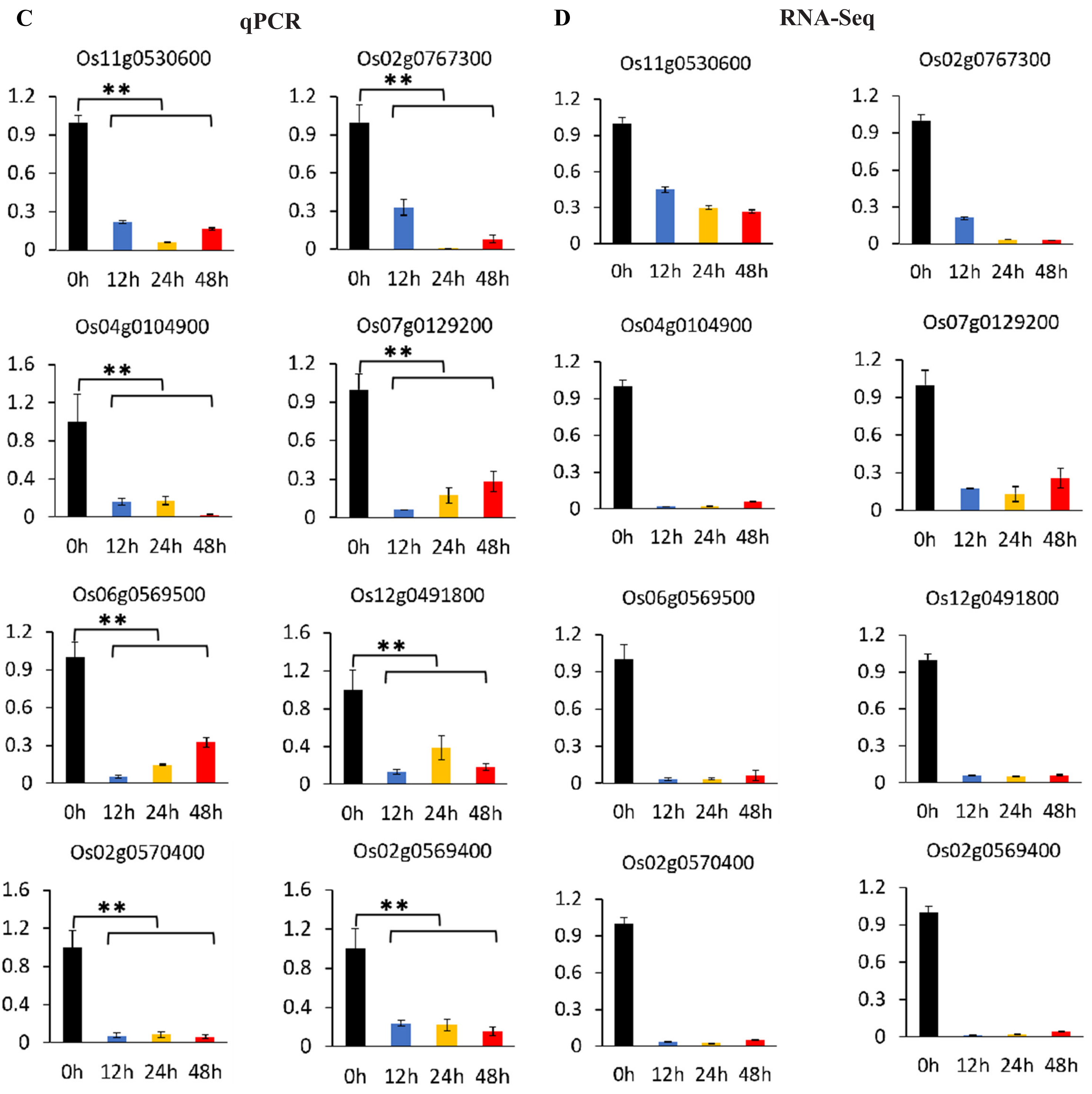
C
qPCR
D
RNA-Seq
Os11g0530600
Os02g0767300
Os04g0104900
Os07g0129200
Os06g0569500
Os12g0491800
Os02g0570400
Os02g0569400
**
0h
12h
24h
48h
0
0.3
0.6
0.9
1.2
0.4
0.8
1.6

Figure S3. Validation of the gene expression profiles obtained by RNA-seq using quantitative polymerase chain reaction (qPCR). Fold-changes in transcript abundance obtained by both qPCR and RNA-seq were presented on the same graph for fourteen genes. (**A**) and (**C**) showed the fold-change determined by PCR, (**B**) and (**D**) showed the fold-change in the Fragments Per Kilobase of transcript sequence per Millions mapped reads (FPKM) obtained by RNA-seq. Os11g0530600, Os02g0767300, Os03g0367200, and Os04g0104900 were involved in flavonoid biosynthesis; Os06g0589600, Os07g0129200, and Os11g0117400 were involved in plant-pathogen interaction; Os06g0569500, Os12g0491800, Os02g0570400, and Os02g0569400 were involved in diterpenoid biosynthesis; and other three genes: Os09g0403300, Os12g0441600, and Os12g0559200. * $p < 0.05$, ** $p < 0.01$.
