# Supplementary material for: Small Brown Planthopper Nymph Infestation Regulates Plant Defenses by Affecting Secondary Metabolite Biosynthesis in Rice
Source: Int J Mol Sci. 2023 Mar 1;24(5):4764. doi: 10.3390/ijms24054764 (PMC10003665; doi:10.3390/ijms24054764)
Supplement: Supplementary file 1 [file ijms-24-04764-s001.zip › Figure S4.pdf]

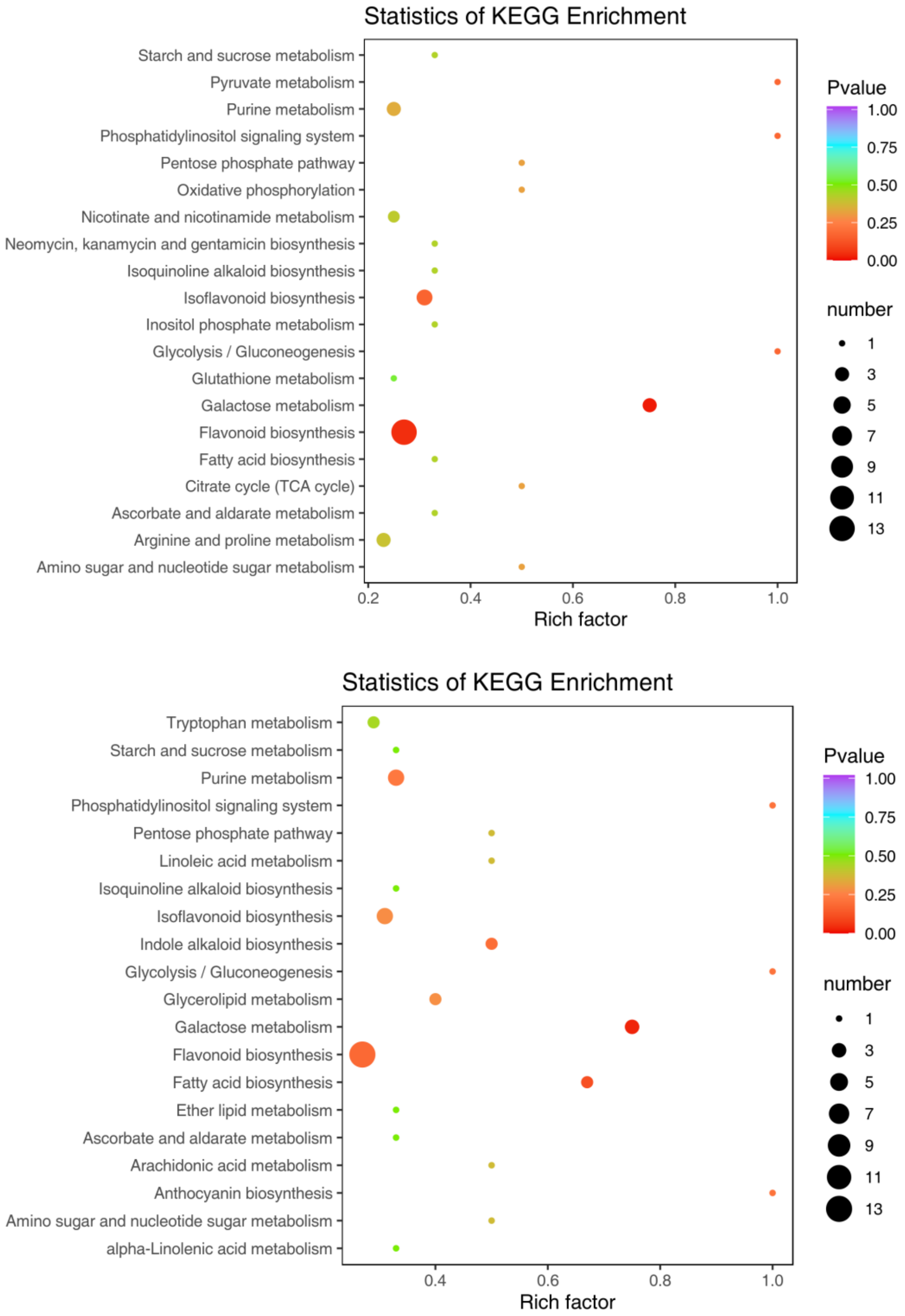


Figure S4. Statistics of Kyoto Encyclopedia of Genes and Genomes (KEGG) pathway enrichment of differentially expressed metabolites in meta24 vs. meta0 group (upper picture) and meta72 vs. meta0 group (bottom picture), respectively. The variable importance in projection (VIP) ≥ 1.0 and the absolute value of $Log_2FC$ (fold-change) ≥ 1.0 are used as the threshold for significantly regulated metabolites.
