# Supplementary material for: Small Brown Planthopper Nymph Infestation Regulates Plant Defenses by Affecting Secondary Metabolite Biosynthesis in Rice
Source: Int J Mol Sci. 2023 Mar 1;24(5):4764. doi: 10.3390/ijms24054764 (PMC10003665; doi:10.3390/ijms24054764)
Supplement: Supplementary file 1 [file ijms-24-04764-s001.zip › Figure S5.pdf]

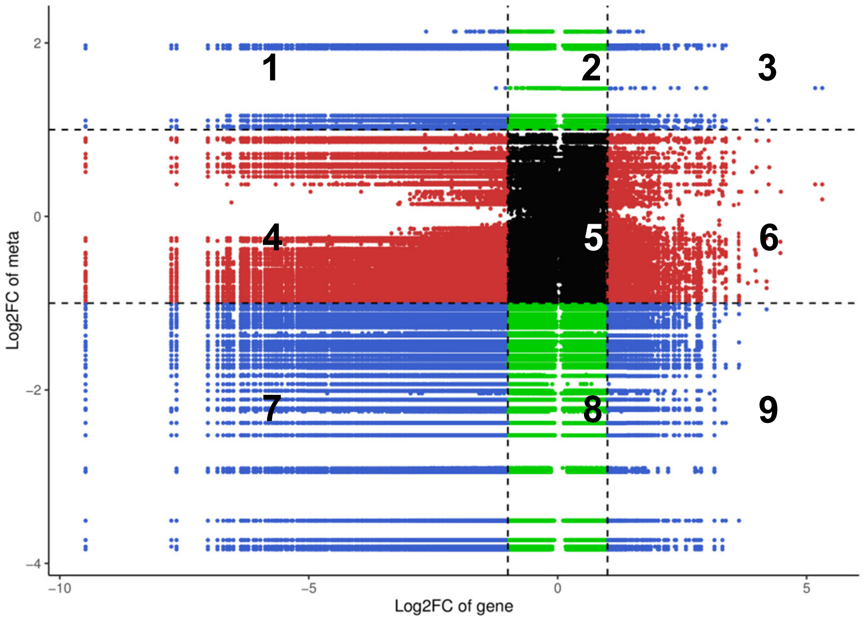

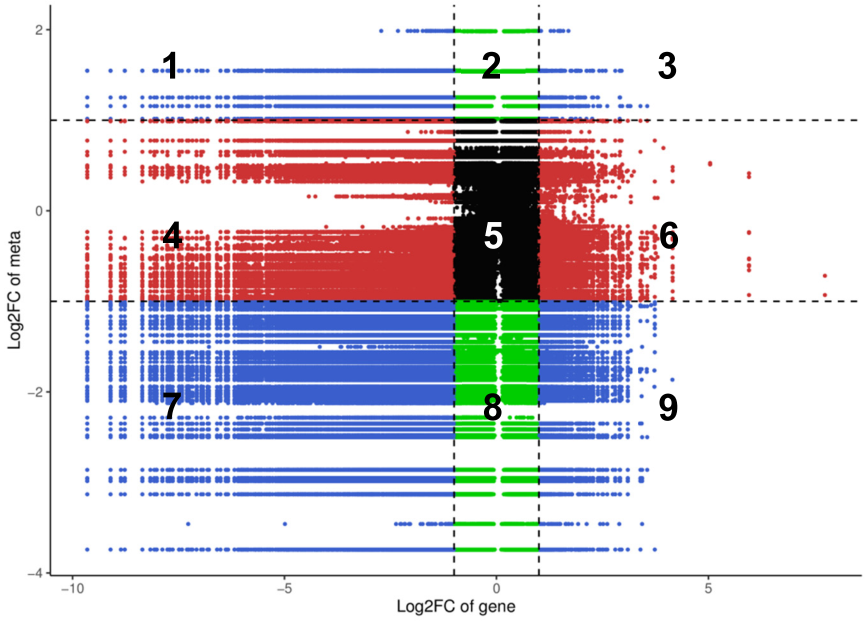

Figure S5. Correlation analysis nine-quadrant diagram showing the association of differentially expressed genes and differentially accumulated metabolites in the meta24 vs. meta0 comparison group (upper picture) and the meta72 vs. meta0 comparison group (bottom picture). The pearson correlation coefficient (PCC) > 0.80 and the *p*-value ≤ 0.05 were chosen and visualized using a nine-quadrant graph. The X-axis represents the $\log_2$FC of genes, and the Y-axis represents the $\log_2$FC of metabolites. From left

to right, from top to bottom, the nine-quadrant diagram was divided into 1 to 9 quadrants used a black dotted line. Quadrant 1 and 9 indicates that the differential expression patterns of genes and metabolites were opposite; quadrant 5 indicate that neither genes nor metabolites were differentially expressed; quadrant 2,4,6 and 8 indicates that metabolites expression unchanged and genes were up or down-regulated, or genes expression were unchanged but metabolites were up or down regulated; quadrant 3 and 7 indicates that the differential expression patterns of genes and metabolites are consistent.
