# Supplementary material for: Small Brown Planthopper Nymph Infestation Regulates Plant Defenses by Affecting Secondary Metabolite Biosynthesis in Rice
Source: Int J Mol Sci. 2023 Mar 1;24(5):4764. doi: 10.3390/ijms24054764 (PMC10003665; doi:10.3390/ijms24054764)
Supplement: Supplementary file 1 [file ijms-24-04764-s001.zip › Figure S6.pdf]

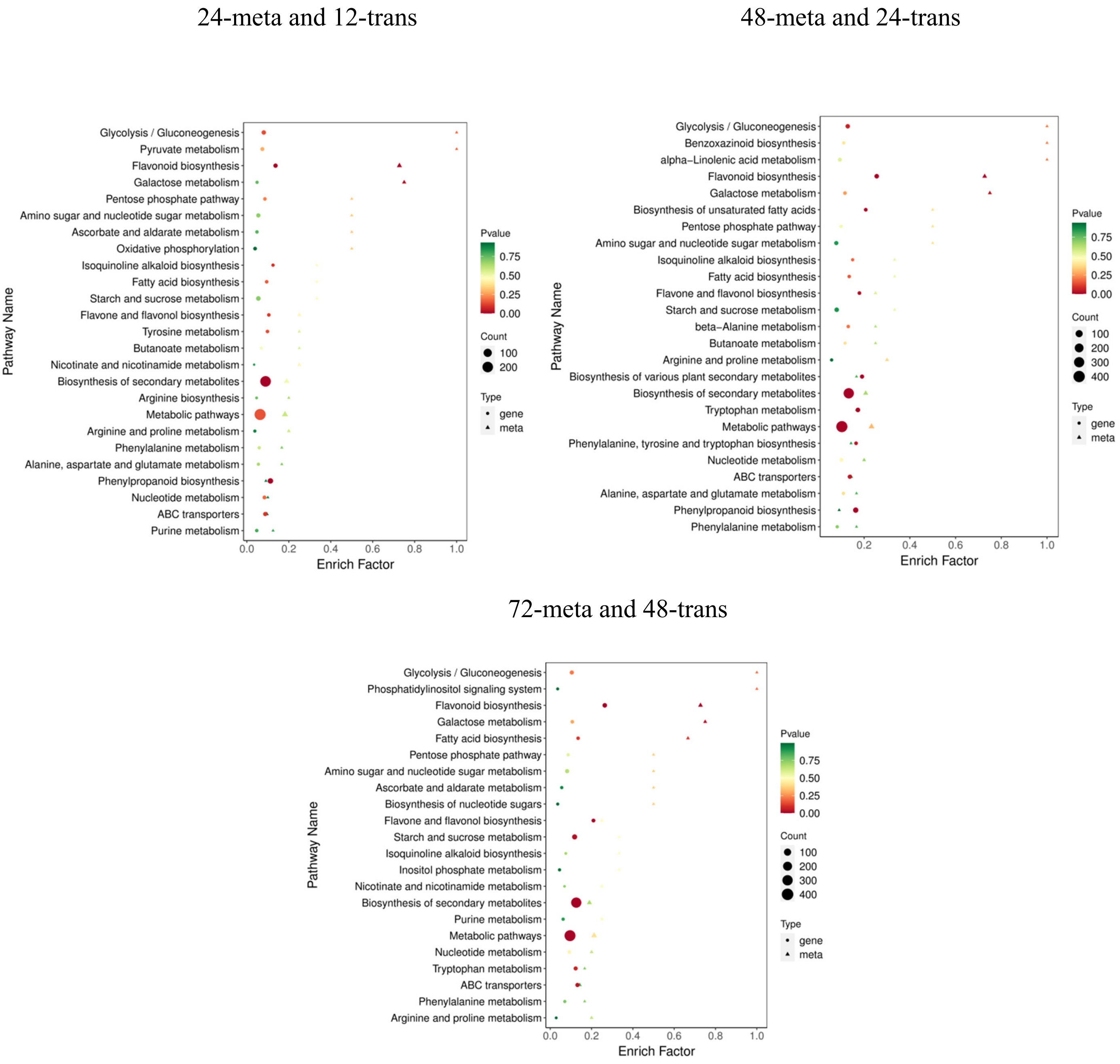

Figure S6. Statistics of Kyoto Encyclopedia of Genes and Genomes (KEGG) pathways co-enriched by transcriptome and metabolome data. Hierarchical cluster analysis (HCA) of all differentially expressed genes and differentially accumulated metabolites between the SBPH-infested (meta24-trans12, meta48-trans24 and meta72-trans48) and uninfested (meta0-trans0) groups. *p*-value ≤ 0.05 and the absolute value of Log2 FC (fold-change) ≥ 1.0 were used as the threshold for significant difference.
