# Supplementary material for: Small Brown Planthopper Nymph Infestation Regulates Plant Defenses by Affecting Secondary Metabolite Biosynthesis in Rice
Source: Int J Mol Sci. 2023 Mar 1;24(5):4764. doi: 10.3390/ijms24054764 (PMC10003665; doi:10.3390/ijms24054764)
Supplement: Supplementary file 1 [file ijms-24-04764-s001.zip › Figure S8.pdf]

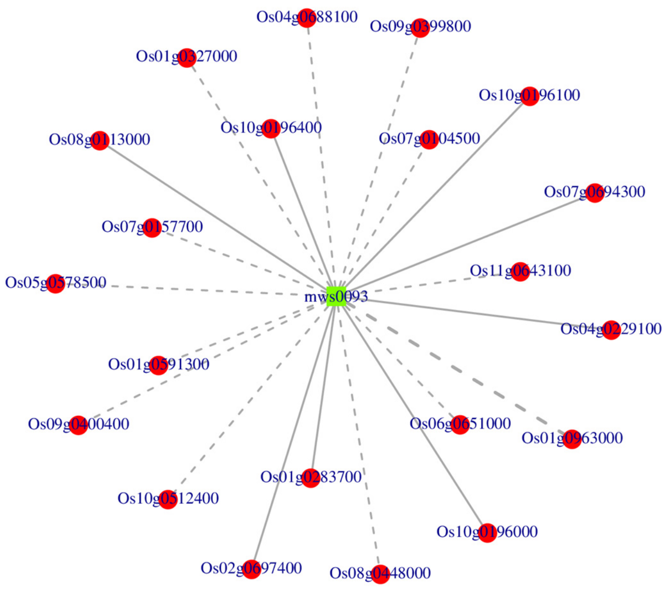

Figure S8. Correlation networks of differentially expressed genes and differentially accumulated metabolites involved in Phenylpropanoid biosynthesis (ko00940). The metabolites are marked with green solid circles and genes are marked with red solid circles. Solid lines represent positive correlations and dashed lines represent negative correlations.
